# Prothrombin complex concentrate vs. fresh frozen plasma in adult patients undergoing heart surgery – a pilot randomised controlled trial (PROPHESY trial)

**DOI:** 10.1111/anae.15327

**Published:** 2020-12-07

**Authors:** L. Green, N. Roberts, J. Cooper, S. Agarwal, S.J. Brunskill, I. Chang, R. Gill, A. Johnston, A. A. Klein, S. Platton, A. Rossi, A. Sepehripour, S. Stanworth, V. Monk, B. O'Brien

**Affiliations:** ^1^ Blizard Institute Barts and the London School of Medicine Queen Mary University of London London UK; ^2^ Department of Haematology Barts Health NHS Trust London UK; ^3^ Department of Cardiac Surgery Barts Health NHS Trust London UK; ^4^ William Harvey Research Institute Barts and the London School of Medicine Queen Mary University of London London UK; ^5^ Department of Anaesthesia Manchester Royal Infirmary Manchester UK; ^6^ Systematic Review Initiative NHS Blood and Transplant Oxford UK; ^7^ Department of Anaesthesia University Hospital Southampton Southampton UK; ^8^ William Harvey Research Institute Barts and The London School of Medicine and Dentistry Queen Mary University of London London UK; ^9^ Department of Anaesthesia Royal Papworth Hospital Cambridge UK; ^10^ Department of Haematology Oxford University Hospitals NHS Foundation Trust Oxford UK; ^11^ Department of Anaesthesia Barts Health NHS Trust London UK; ^12^ NHS Blood and Transplant Oxford UK; ^13^ Outcomes Research Consortium, Cleveland Clinic OH USA

**Keywords:** bleeding, cardiac surgery, FFP, PCC, randomised trial

## Abstract

There is equipoise regarding the use of prothrombin complex concentrate vs. fresh frozen plasma in bleeding patients undergoing cardiac surgery. We performed a pilot randomised controlled trial to determine the recruitment rate for a large trial, comparing the impact of prothrombin complex concentrate vs. fresh frozen plasma on haemostasis (1 h and 24 h post‐intervention), and assessing safety. Adult patients who developed bleeding within 24 h of cardiac surgery that required coagulation factor replacement were randomly allocated to receive prothrombin complex concentrate (15 IU.kg^−1^ based on factor IX) or fresh frozen plasma (15 ml.kg^−1^). If bleeding continued after the first administration of prothrombin complex concentrate or fresh frozen plasma administration, standard care was administered. From February 2019 to October 2019, 180 patients were screened, of which 134 (74.4% (95%CI 67–81%)) consented, 59 bled excessively and 50 were randomly allocated; 25 in each arm, recruitment rate 35% (95%CI 27–44%). There were 23 trial protocol deviations, 137 adverse events (75 prothrombin complex concentrate vs. 62 fresh frozen plasma) and 18 serious adverse events (5 prothrombin complex concentrate vs. 13 fresh frozen plasma). There was no increase in thromboembolic events with prothrombin complex concentrate. No patient withdrew from the study, four were lost to follow‐up and two died. At 1 h after administration of the intervention there was a significant increase in fibrinogen, Factor V, Factor XII, Factor XIII, α_2_‐antiplasmin and antithrombin levels in the fresh frozen plasma arm, while Factor II and Factor X were significantly higher in the prothrombin complex concentrate group. At 24 h, there were no significant differences in clotting factor levels. We conclude that recruitment to a larger study is feasible. Haemostatic tests have provided useful insight into the haemostatic changes following prothrombin complex concentrate or fresh frozen plasma administration. A definitive trial is needed to ascertain the benefits and safety for each.

## Introduction

Major bleeding that requires blood transfusion during cardiac surgery is associated with significant morbidity and mortality [1]. Standard care in the UK for replacement of clotting factors during bleeding in patients who are not taking vitamin K antagonists is fresh frozen plasma (FFP) [2]. The use of FFP in patients undergoing cardiac surgery is approximately 20–30% depending on the surgical procedure performed [3]. Evidence for the use of FFP in bleeding patients is lacking, and this was highlighted in a Cochrane systematic review [4]. Furthermore, FFP transfusion is associated with adverse effects such as fluid overload, acute pulmonary hypertension, transfusion‐transmitted infections and immunomodulatory side‐effects. Hence, haemostatic agents such as prothrombin complex concentrate (PCC) are used for management of bleeding in this setting, due to the potential advantages of a higher concentration of clotting factors compared with FFP, faster administration and smaller volume, meaning that it can be administered quickly to patients susceptible to volume overload, resulting in less haemodilution. There have been no randomised controlled trials that have compared the clinical efficacy and safety of PCC vs. FFP in bleeding patients undergoing cardiac surgery and who are not on vitamin K antagonists. A recent systematic review of PCC use in cardiac surgery (861 adult patients) concluded that PCC was safe in this setting, and that its administration was associated with reduced blood transfusion (OR 2.22; 95%CI 1.45–3.40), although there were no differences in other outcomes. A trend towards increased risk of renal replacement therapy was observed in the PCC group (OR 0.41; 95%CI 0.16–1.02) [5]. As part of preparing for an appropriately powered randomised controlled trial comparing the efficacy and safety of PCC with FFP in cardiac surgical patients who are bleeding, we performed a single‐site pilot randomised controlled trial to determine the recruitment rate for a large trial, comparing the impact of PCC and FFP on the haemostatic capacity of bleeding patients, and assess trial procedures, protocol compliance/violation and safety outcomes.

## Methods

The trial methodology and reason for its design have previously been published [6]. Inclusion criteria were adult patients (≥ 18 years), who were able to give consent and were undergoing elective or non‐elective cardiac surgery (excluding procedures listed in online Supporting Information, Appendix S1) [6]. Written informed consent was obtained from all patients before surgery. For participants who were randomly allocated, clinical data were collected prospectively for up to 90 days after surgery (see online Supporting Information, Appendix S1), whereas for participants who were enrolled, but not randomly allocated (because they did not bleed), clinical data were collected prospectively for up to 24 h after surgery. Enrolled participants were randomly allocated by the transfusion laboratory to receive either PCC or FFP if they developed active bleeding within 24 h of surgery, at the point when the clinician requested FFP for the treatment of bleeding. An FFP transfusion would be requested if thromboelastography was abnormal (see the algorithm in online Supporting Information, Appendix S1), and if thromboelastography or clotting test results were not available, it was administered in a 1:2 ratio with red cells, in line with national guidance [2]. Randomisation was by allocating participants in a 1:1 ratio to receive PCC or FFP using block randomisation, with block size varied randomly to ensure balance of treatments. The algorithm was written by the study statistician using the ralloc command in Stata. Randomisation occurred via a web‐based electronic database (REDCap) [7] for the first 5 months of the trial and was switched to manual randomisation envelopes for the next 3 months. Laboratory staff found paper randomisation easier and simpler to use during an emergency and the pilot study was designed to be responsive to learning and improvement ideas while the trial progressed.

Patients were randomly allocated to one of two groups: an intervention arm (four‐factor PCC; Octaplex, Octapharma Ltd., Manchester, UK) or a standard arm (FFP). Both products were stored in laboratories, but PCC was reconstituted at the bed‐side. There is currently no agreed dose for PCC for the management of acquired bleeding disorders not related to vitamin K antagonists. The European Society of Anaesthesiology guidelines recommend a dose of 20–30 IU.kg^−1^ [8] and other observational studies have used doses ranging from 1000 IU (or 11.5 IU.kg^−1^) [9] to 1500 IU for three‐factor PCC [10]. Based on these studies, and the current recommended dose for FFP in the UK (15 ml.kg^−1^), the following dose schedule was used for PCC in this study based on Factor IX level (doses were rounded up to avoid wastage): 500 IU if the patient's weight was < 60 kg; 1000 IU if 61–90 kg; and 1500 IU if > 90 kg. If bleeding continued after administration of the first PCC dose, the patient received standard care with FFP; therefore, no further PCC was administered to any patient. The dose of FFP used in this study was 15 ml.kg^−1^ and, based on the average volume of one FFP unit being 270 ml [11], we rounded up the doses to reduce wastage to 3 units if the patient's weight was ≤ 60 kg, 4 units if 61–90 kg and 5 units if > 90 kg.

The primary outcome was the proportion of eligible patients who consented and received the intervention within 24 h of surgery. Two months after the trial started, an amendment was made to the protocol to clarify the definition of ‘recruitment’ as those individuals who consented, were randomly allocated and received the intervention or control. For this trial to be successful, it was pre‐specified that ≥ 30% of eligible patients must have agreed to participate, and ≥ 30% of consented patients must have been randomly allocated and received the intervention within 24 h of surgery in order to proceed to the full trial. Secondary outcomes included: proportion of patients where there was protocol adherence and protocol violation; difference in haemostatic capacity; time to administration of study drug; and safety up to 90 days after surgery (see definitions in online Supporting Information, Appendix S1). To assess haemostatic capacity, blood samples were taken at three time‐points: before the intervention and during bleeding; within 1 h of the intervention being completed; and 24 h after the intervention (for sample handling, see online Supporting Information, Appendix S1). All samples were analysed by a biomedical scientist who was blinded to the patient group. The following tests were performed in accordance with the laboratory standard operating procedures: prothrombin time; activated partial thromboplastin time; Clauss fibrinogen; D‐dimer; heparin levels (anti‐10a and anti‐2a); Factors II, V, VII, VIII, IX, X, XI, XII, XIII; von Willebrand antigen and activity; high molecular weight kininogen; prekallikrein; C1‐inhibitor; antithrombin activity; protein C activity; free protein S antigen; α_2_‐antiplasmin; plasminogen; tissue plasminogen activator; tissue factor activatable fibrinolysis inhibitor; prothrombin Factor I and II; thrombin‐antithrombin complex; plasmin‐antiplasmin complex; soluble endothelial protein C Receptor; thrombomodulin; tissue factor; and thrombin generation.

Sample size was based on estimating a consent rate of 30% of an expected 638 eligible participants over a 15‐month period, with a 95%CI of ±3.5%. We also estimated that 30% of an expected 191 consented patients would go on to develop bleeding during, or within 24 h of, surgery that required FFP transfusion, allowing us to estimate a proportion of 30% with a 95%CI of ±6.5%. Based on the above, approximately 57 patients would be randomly allocated within 15 months, giving an expected final sample size of 51 patients completing the study after allowing for a 10% drop out or loss to follow‐up. As this was a pilot study, no hypothesis testing was undertaken.

## Results

Recruitment started in February 2019 and the last patient was randomly allocated on 28 October 2019. Final follow‐up data were collected on 29 January 2020.

**Figure 1:**
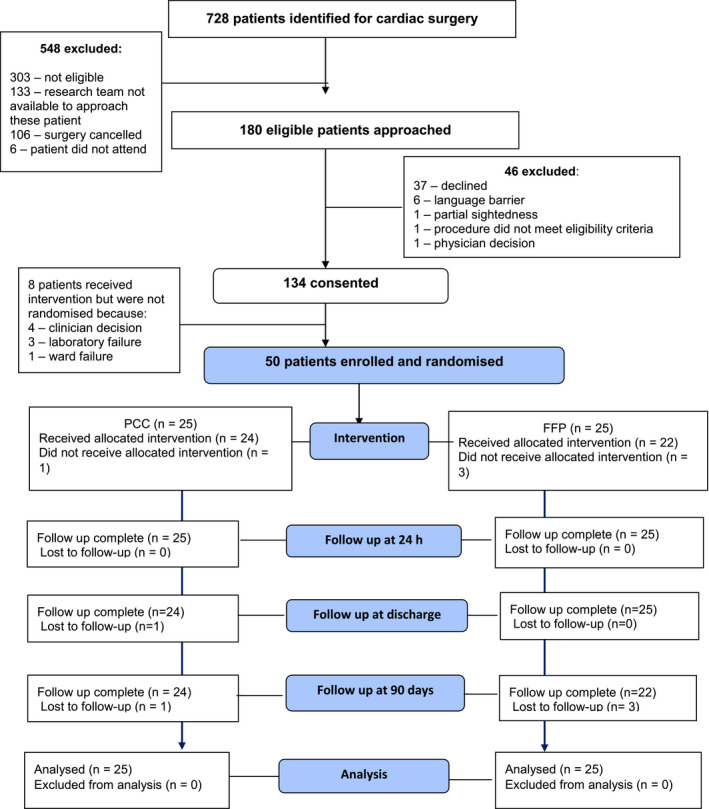
Study flow diagram showing patients who were identified for inclusion in the study, enrolled, randomly allocated and analysed. PCC, prothrombin complex concentrate; FFP, fresh frozen plasma.

Of the 727 patients who underwent cardiac surgery during the study period, 180 (24.8%) were eligible, 134 participants consented and were enrolled in the trial (consent rate of 74.4% (95%CI 67.4–80.6%). Reasons for not enrolling eligible patients are given in Fig. 1, with the main reasons being declined consent (n = 37) and language barrier (n = 6). Out of 134 patients who enrolled in the trial, 59 patients bled within 24 h, and of the latter 50 were appropriately randomly allocated (25 FFP and 25 PCC), of which 47 received the intervention, giving a recruitment rate of 35.1% (95%CI 27.0–43.8%). Of the 25 subjects allocated to PCC and 25 allocated to FFP, no‐one withdrew from the trial but four were lost to follow‐up at 90 days. Reasons for loss to follow‐up were patients returned to their homes abroad and were not able to be contacted (n = 2), and patients were not reachable by telephone following several attempts (n = 2). The intention to treat group included 25 subjects in each group and per‐protocol analysis included 21 subjects for both PCC and FFP arms. Reasons for excluding eight patients from per‐protocol analysis were due to protocol violations which included: three patients randomly allocated to FFP did not receive the intervention because bleeding had ceased by the time the products arrived; three had research blood sample violation; one was randomly allocated with a non‐sequential envelope; and one randomly allocated to PCC had already received FFP. Baseline characteristics for enrolled and randomly allocated patients are presented in Table 1. Of the 50 randomly allocated patients, three in the FFP arm did not receive the intervention because they had stopped bleeding by the time the products had been issued. Of the remaining 47, completion of the intervention was documented for all subjects. Overall protocol adherence was achieved for 42/50 patients (84%) with no difference between groups. There were 23 trial protocol deviations relating to randomisation/intervention (n = 14); documentation (n = 5); research blood sample collection (n = 3); and pharmacy (n = 1). Of the 14 deviations relating to randomisation/intervention, nine patients bled excessively but were not randomly allocated (eight received an intervention and one did not as bleeding had stopped when the products arrived), and reasons for these were laboratory failure (n = 3); clinician decision not to randomly allocated patients (n = 4); research team error (n = 1); and ward failure (n = 1).

**Table 1:** Characteristics of patients enrolled in the study but not randomly allocated or who were enrolled in the study and randomly allocated. Values are median (IQR [range]), number (proportion) or mean (SD).

	Enrolled but not randomly allocated n = 84	Enrolled and randomly allocated n = 50	
		PCC n = 25	FFP n = 25
Age; years	68 (57–76 [25–85])	69 (63–73 [28–85])	66 (57–74 [ 41–88])
Sex; male	53 (63.1%)	16 (64.0%)	16 (64.0%)
Ethnicity			
White	66 (85.7%)	21 (95.5%)	20 (90.9%)
Asian	6 (7.8%)	1 (4.6%)	1 (4.6%)
African	4 (5.2%)	0 (0%)	1 (4.6%)
Mixed	1 (1.3%)	0 (0%)	0 (0%)
Weight; kg	78.0 (17.3)	76.1 (19.7)	80.5 (16.2)
Height; cm	170 (10)	167 (10)	168 (11)
BMI; kg.m^−2^	27 (6)	27 (6)	29 (5)
EuroSCORE; %	2 (1–4 [1–18])	4 (2–6 [1–15])	3 (2–5 [1–46])
Medical history			
Diabetes mellitus	13 (16.5%)	6 (24.0%)	6 (24.0%)
Hypertension	50 (63.3%)	19 (76.0%)	13 (52.0%)
Angina	12 (15.2%)	6 (24.0%)	6 (24.0%)
Previous PCI	2 (2.5%)	3 (12.0%)	2 (8.0%)
Previous cardiac surgery	4 (5.1%)	2 (8.0%)	1 (4%)
Elective surgery	71 (89.9%)	19 (76.0%)	20 (80.0%)
Procedure			
Valve only	34 (40.5%)	5 (20.0%)	7 (28.0%)
Major aortic valve only	3 (3.6%)	2 (8.0%)	0 (0.0%)
CABG + valve	17 (20.2%)	6 (24.0%)	5 (20.0%)
Complex/combined procedure	30 (35.7%)	12 (48.0%)	13 (52.0%)
Medication			
Antiplatelet	1 (1.2%)	7 (28.0%)*	7 (28.0%)^†^
Anticoagulant	7 (8.3%)	9 (36.0%)	7 (28.0%)
EQ5D index score	0.9 (0.7–0.9 [0.0–1.0])	0.9 (0.8–1.0 [0.3–1.0])	0.9 (0.7–1.0 [0.4–1.0])
Laboratory tests at screening (pre‐surgery)			
Haemoglobin; g.l^−1^	133 (124–143 [104–167])	130 (124–141 [109–152])	135 (121–145 [95–166])
Platelets; ×10^9^.l^−1^	227 (184–271 [107–423])	239 (194–259 [139–314])	233 (176–296 [123–366])
PT; s	11 (11–12 [10–18])	11 (11–12 [10–17])	11 (11–12 [10–13])
APTT; s	26 (24–28 [21–36])	27 (25–29 [21–35])	25 (23–27 [21–67])

PCC, prothrombin complex concentrate; FFP, fresh frozen plasma; PCI, percutaneous coronary interventions; EQ5D, health questionnaire; CABG, coronary artery bypass grafts; PT, prothrombin time; APTT, activated partial thromboplastin time.

* 7 PCC patients were taking antiplatelet medication (4 aspirin, 3 clopidogrel).

^†^ 7 FFP patients were taking aspirin and of these one was on both aspirin and clopidogrel.

Median (IQR [range]) time from contacting the laboratory to first administration of intervention was 63 (36–108 [2–255]) min in the PCC group and 72 (40–114 [30–146]) min in the FFP group. Ninety‐day follow‐up data were collected for 92% of randomly allocated patients. Missing data for clinical outcomes at 90 days were below the 20% level set for progression to the full trial for all outcomes, except quality of life data which were 34% (95%CI 21.2–48.8%). Transfusion requirements at 24 h, 7 days and 30 days are shown in Table 2 and were similar between the two groups. Median (IQR [range]) time from intervention to chest closure was 80 (45–195 [0–610]) min in the PCC group and 105 (45–173 [10–328]) min in the FFP group (difference of 25 min, 95%CI −65.8 to 115.8) and median (IQR [range]) volume of chest drain losses at 24 h was 525 (375–838 [180–2200]) ml in the PCC group and 575 (425–750 [300–1425]) ml in the FFP group.

**Table 2:** Intervention doses, blood loss and blood components administered to patients included in the study. Values are median (IQR [range]) or number (proportion).

	Intention to treat population		Per protocol	
	PCC n = 25	FFP n = 25^‡^	PCC n = 21	FFP n = 21^‡^
Dose of intervention				
Octaplex; IU*	1000 (1000–1000 [0–1500])		1000 (1000–1000 [500–1500])	
FFP; units		4 (4–5 [3–5])		4 (4–5 [3–5]]
Blood loss in chest drains at 24 h; ml	525 (375–838 [180–2200)]	575 (425–750 [300–1425])	475 (375–850 [275–2200])	575 (425–825 [325–1425])
Blood components received at 24 h; units^†^				
Total	4 (2–7 [1–16])	3 (1–6 [0–31])	4 (2–7 [1–16])	3 (1–6 [0–31])
Red cells	2 (1–3 [0–6])	2 (0–4 [0–12])	2 (1–3 [0–6])	2 (0–4 [0–12])
FFP	0 (0–0 [0–6])	0 (0–0 [0–10])	0 (0–0 [0–6])	0 (0–0 [0–10])
Platelets	1 (1–2 [0–4])	1 (0–2 [0–6])	1 (1–2 [0–4])	1 (0–2 [0–6])
Cryoprecipitate	1 (0–1 [0–3])	0 (0–2 [0–3])	0 (0–1 [0–3])	0 (0–2 [0–3])
Blood components received at 7 days; units^†^				
Total	6 (3–8 [1–16])	4 (2–7 [0–37])	5 (3–8 [1–16])	4 (2–7 [1–37])
Red cells	3 (2–4 [0–9])	2 (1–4 [0–16])	3 (2–4 [0–9])	2 (1–4 [0–16])
FFP	0 (0–0 [0–6])	0 (0–0 [0–12])	0 (0–0 [0–6])	0 (0–0 [0–12])
Platelets	1 (1–2 [0–5])	1 (0–2 [0–6])	1 (1–2 [0–5])	1 (0–2 [0–6])
Cryoprecipitate	1 (0–2 [0–3])	0 (0–2 [0–3])	0 (0–1 [0–3])	0 (0–2 [0–3])
Blood components received at 30 days; units^†^				
Total	6 (3–9 [1–21])	5 (2–7 [0–37])	5 (3–8 [1–21])	5 (2–7 [1–37])
Red cells	3 (2–5 [0–13])	2 (1–4 [0–16])	3 (2–4 [0–13])	2 (2–4 [0–16])
FFP	0 (0–0 [0–6])	0 (0–0 [0–12])	0 (0–0 [0–6])	0 (0–0 [0–12])
Platelets	1 (1–2 [0–5])	1 (0–2 [0–6])	1 (1–2 [0–5])	1 (0–2 [0–6])
Cryoprecipitate	1 (0–2 [0–3])	0 (0–2 [0–3])	0 (0–1 [0–3])	0 (0–2 [0–3])
Haemostatic agents administered within 24 h of surgery				
Recombinant factor VIIa	0	0	0 (0%)	0 (0%)
Fibrinogen concentrate	0	0	0 (0%)	0 (0%)
Cell salvage	17 (68%)	18 (72%)	14 (67%)	15 (71%)
Time from intervention to chest closure; min	80 (45–195 [0–610])	105 (45–173 [10–328])	75 (40–170 [0–250])	105 (45–173 [10–328])

PPC, prothrombin complex concentrate; FFP, fresh frozen plasma.

* 1 patient randomly allocated to PCC received 5 units of FFP.

^†^ Excludes FFP intervention.

^‡^ 3 patients randomly allocated to FFP did not receive intervention.

**Figure 2:**
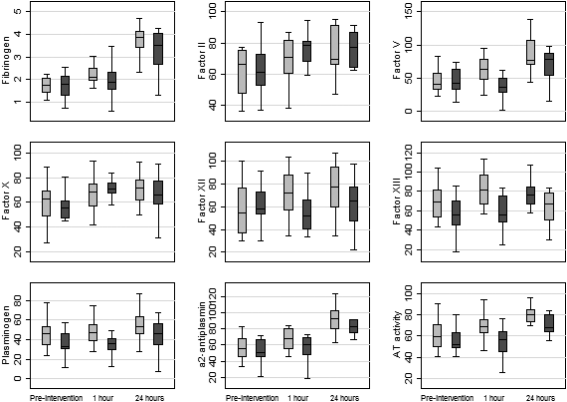
Clotting factor results pre‐intervention (and during bleeding) at 1 h and 24 h after intervention for both arms. Line is median, box is IQR and whiskers are range. Grey, fresh frozen plasma; dark grey, prothrombin complex concentrate.

Results of all haemostatic assays are presented as changes from baseline at 1 h and 24 h after intervention (online Supporting Information, Appendix S1), and Fig. 2 describes the results of the assays that changed significantly. One hour after administration of the intervention, there was a significant increase in fibrinogen, Factors V, XII and XIII, α_2_‐antiplasmin and antithrombin levels in the FFP group, while Factors II and X were significantly higher in the PCC group. Plasminogen levels were significantly higher in the FFP group at both time‐points (1 h and 24 h). At 24 h, there were no significant differences in other factors.

Two patients (4%), one from each group, died within 90 days with the causes being congestive cardiac failure and heart valve incompetence (Table 3). Average number of days alive and out of hospital by 90 days was 80 days for both arms, and median (IQR [range]) number of days spent in hospital were 12 (7–14 [5–90]) days for patients in the PCC group and 10 (6–14 [2–90]) days for patients in the FFP group. A total of 59 patients bled excessively, 55 received a study intervention and all were included in the safety analysis (Table 4). For patients receiving an intervention, there were 137 adverse events (75 in the PCC group vs. 62 in the FFP group) and 18 serious adverse events (five out of four patients receiving PCC vs. thirteen out of nine patients receiving FFP). Most serious adverse events in the FFP group were due to re‐hospitalisation (9/13) not related to their intervention and one patient was re‐hospitalised three times within 90 days of surgery. No transfusion reactions were reported.

**Table 3:** Event rates up to 90 days postoperatively for patients included in the study. Values are number (proportion) or median (IQR [range]).

	Intention to treat population		Per protocol population	
	PCC group n = 25	FFP group n = 25	PCC group n = 21	FFP group n = 21
Deaths	1 (4.2%)	1 (4.2%)	1 (5%)	1 (5%)
Resternotomy	3 (12%)	3 (12%)	2 (9.5%)	3 (14.3%)
Infection	4 (16%)	5 (20%)	3 (12%)	5 (20%)
Chest drainage at 24 h; ml*	525 (375–838 [180–2200])	575 (425–750 [300–1425])	475 (375–850 [275–2200])	575 (425–825 [325–1425])
Organ failure	2 (8%)	2 (8%)	2 (9.5%)	1 (4.8%)
Haemodialysis	3 (12%)	4 (16%)	3 (14.3%)	3 (14.3%)
ICU or HDU stay; days^†^	4 (3–6 [0–55])	4 (3–5 [2–25])	4 (3–6 [0–55])	4 (3–5 [2–25])
Duration of hospital stay; days	9 (7–13 [5–90])	7 (6–13 [2–90])	9 (7–13 [5–90])	7 (6–12 [2–90])
Days alive and out of hospital at 90 days	80 (77–83 [0–90])	81 (76–84 [0–88])	82 (77–83.5 [0–90])	81 (71–84 [0–88])
Days spent in hospital at 90 days	12 (7–14 [5–90])	10 (6–14 [2–90])	10 (7–14 [5–90])	9 (6–19 [2–90])

PCC, prothrombin complex concentrate; FFP, fresh frozen plasma; ICU, intensive care unit; HDU, high dependency unit.

* Not available for 1 PCC and 3 FFP patients.

^†^ All patients went to ITU apart for one patient in the PCC group.

**Table 4:** Safety analysis for patients included in the study. Values are number or number (proportion).

	PCC group n = 29	FFP group n = 26
Total number of adverse events	75	62
Patients with adverse events	22 (76%)	19 (73%)
Total number of serious adverse events	5	13
Patients with serious adverse events	4 (14%)	9 (35%)
Types of serious adverse events*		
Resulting in death	1	1
Heart failure	1	
Heart valve incompetence		1
Life‐threatening	2	2
Heart failure^†^		1
Mesenteric artery thrombosis	1	
Multiple organ failure	1	
Pulseless electrical activity^†^		1
Re‐hospitalisation or prolonged hospitalisation	1	9
Abdominal pain^‡^		1
Chest pain^‡^		1
Malaise^‡^		1
Heart failure		1
Hypotension		1
Haemothorax^§^		1
Pleuritic pain		1
Raised INR	1	
Stroke^§^		2
Persistent or significant disability	1	1
Spinal cord ischaemia	1	
Stroke		1

PCC, prothrombin complex concentrate; FFP, fresh frozen plasma; INR, international normalised ratio.

* Thromboembolic events are classified as serious adverse effects, there were no venous thromboses in either arm.

^†^ Two life‐threatening serious adverse events were for one patient in the FFP arm.

^‡^ Three hospital re‐admissions were from the same patient in the FFP arm.

^§^ Haemothorax resulted in re‐admission, and a stroke during that admission, led to prolonged hospitalisation for one patient.

We updated the most recent PCC systematic review [5] to assess the two safety outcomes (mortality and stroke) from this study, those from the review (all observational studies), and addition of any other studies (we identified one other observational study [12]) that have been published since the publication of the review (the search was updated on 27 June 2020). We observed no difference in all‐cause mortality or stroke between people receiving PCC and those receiving FFP (online Supporting Information, Appendix S1, Figure S1).

## Discussion

We have demonstrated that it is feasible to enroll patients into a randomised controlled trial comparing transfusion of PCC with FFP in adult patients not taking vitamin K antagonists who develop bleeding while undergoing cardiac surgery. In our trial, both primary outcome criteria were met (74.4% consented and 35.1% randomised and treated), establishing that a larger trial, powered to detect a difference of effect, would be feasible. Our trial protocol was integrated into routine clinical care, allowing for randomisation to continue outside normal working hours and at weekends (through the transfusion laboratory) when the research team were not on site (for the large trial we will use Sealed Envelope™, https://www.sealedenvelope.com, which is an easy online system to randomly allocate patients). This facilitated rapid recruitment (5 months ahead of schedule), and likely cost savings if the same model is used in a definitive trial. The protocol allowed for transfusion scientists to randomly allocate patients at the point when clinicians requested FFP, and was combined with targeted training sessions with clinicians, minimising any risks of perceived delay to obtaining trial interventions and thereby contributing to low rates of protocol violations. Other randomised controlled trials in the same setting of cardiac surgery have in some cases reported 50% violation rates [13], and this is partly due to study design, which in our trial sought to align interventions with clinicians' judgement on managing bleeding, rather than using a protocol‐driven approach.

A number of learning points for future studies were identified. Median time from clinicians contacting the laboratory to first administration of the products was 63 min for the PCC group and 72 min for the FFP group, although we had expected a quicker time for PCC given there is no need for thawing, and reconstitution can start immediately (in our institution, the thawing time for four units of FFP is approximately 15 min) [14]. Our study did not explore reasons for this, and we recognise the limitations as a single centre study. In most UK hospitals, PCC and FFP are stored in transfusion laboratories, so that their use can be monitored closely in line with UK blood safety and quality regulation. In a large trial, one option would be to explore storing the PCC closer to clinical areas, and the same could apply to FFP, which can now be stored thawed for up to 5 days in the remote blood fridge near clinical areas [15]. In a pilot randomised controlled trial in the trauma setting, which had as its primary objective the feasibility of delivering fibrinogen concentrate within 45 min of hospital admission in bleeding patients, the researchers failed to meet the pre‐defined target of 90% compliance (only 69% achieved this), even though study intervention packs were held in the emergency department. There is a need to understand barriers and enablers to timely delivery of haemostatic agents and in the large trial we will monitor this closely at all participating sites.

A secondary objective of this trial was to compare the impact of PCC and FFP on the haemostatic capacity of bleeding patients. For a range of haemostatic factors, we identified a more balanced increase in clotting factors with FFP compared with PCC. One hour after the intervention, we found a significant increase in procoagulants (fibrinogen, Factors V, XII and XIII), anticoagulants (antithrombin) and fibrinolytic factors (plasminogen and α_2_‐antiplasmin) in the FFP arm compared with the PCC arm. All these factors are lacking in PCC. At 1 h post‐intervention, the increases in Factors II and X (compared with pre‐intervention) were greater in the PCC arm than the FFP arm, but this was not the case for Factors VII and IX. This is likely to be due to Factor VII having the shortest half‐life among the four factors present in PCC, and the fact that the PCC dose for this study was based on Factor IX levels, indicating that the calculated dose for PCC, as far as Factor IX is concerned, is comparable to the FFP dose. At 24 h after the intervention, changes in the four clotting factors were not significant between the two groups. The clinical implications of these changes in haemostatic factors are unclear, and limited by the small sample size. Factor V is essential in generating thrombin [16] which, together with fibrinogen and Factor XIII, plays a crucial role in formation of stabilised cross‐linked fibrin that will help with stopping bleeding. Unlike single clotting factor deficiency bleeding settings, following cardiac surgery bleeding patients lose all their clotting factors and thus it might be sensible to replace all lost factors in order to optimise haemostasis. Our data show that this balance is better achieved with FFP. A recent multinational observational study that compared clinical outcomes of bleeding patients who received PCC compared with FFP showed that the PCC cohort received significantly more fibrinogen concentrate (43% vs.15%) and cryoprecipitate (3.4% vs. 0.5%) than FFP patients, but not red cell transfusions [12].

Antithrombin attenuates thrombin activity and its generation produces thrombin‐antithrombin complexes, while plasminogen is the proenzyme of plasmin (regulated by α_2_‐antiplasmin), the primary target of which is the degradation of cross‐linked fibrin, with the end‐product being dimeric D‐domains (or D‐dimers) [17]. Theoretically, achieving a balance in the procoagulant, anticoagulant and fibrinolytic systems is the aim in managing a bleeding patient. It should be noted in this study, that the results of all global markers for in‐vitro thrombin generation (thrombin generation, Prothrombin factor 1.2 and thrombin‐antithrombin complexes) and fibrinolytic system (D‐dimer) were no different between the PCC and FFP arms at both 1 h and 24 h after surgery, indicating that, as far as global tests are concerned, this balance appears to be about right.

The interpretation of haemostatic changes needs to be made based on the doses used in our trial. The recommended dose for FFP in the UK is based on one observational study in non‐bleeding patients, which reported a dose‐dependent relationship in the main clotting factor levels with higher doses (33.5 ml.kg^−1^) vs. standard doses of FFP (12.2 ml.kg^−1^) [18]. There is no accepted dose for PCC for treatment of bleeding patients who are not receiving vitamin K antagonists. Another on going pilot randomised controlled trial in cardiac surgery (FARES, NCT04114643) is testing a different dose compared with the dose we used in our trial.

No safety concerns were raised in our pilot trial. There is a long history of concerns regarding thrombotic events in association with PCC use, but this was not borne out in our trial when compared with FFP use. We updated the search report for the two safety outcomes (mortality and stroke) using the recent systematic review of PCC use in cardiac surgery and found no suggestion for increased rates of these adverse events.

In conclusion, our pilot study confirms that it is feasible to conduct a larger trial in the future. The doses selected for PCC and FFP in our trial appear well balanced as far as global haemostatic assays are concerned, but it remains to be determined whether these doses translate into different clinical benefits and safety for each of the interventions.

## Supporting information


**Appendix S1.** Supplemental material on exclusion criteria, clinical outcome measures and transfusion algorithms.
