# Supplementary material for: Prothrombin complex concentrate vs. fresh frozen plasma in adult patients undergoing heart surgery – a pilot randomised controlled trial (PROPHESY trial)
Source: Anaesthesia. 2020 Dec 7;76(7):892–901. doi: 10.1111/anae.15327 (PMC8246985; doi:10.1111/anae.15327)
Supplement: Supplementary file 1 — Appendix S1. Supplemental material on exclusion criteria, clinical outcome measures and transfusion algorithms. [file ANAE-76-892-s001.docx]

**Appendix S1**

- 1. Exclusion Criteria

Unable to consent, patients refusing blood transfusion for any reason, first time isolated coronary artery bypass grafts (CABG), first time isolated aortic valve replacement (excluding active endocarditis), thoraco-abdominal surgeries, minor surgeries that do not involve cardiopulmonary bypass, use of warfarin within three days, use of direct oral anticoagulants (i.e. dabigatran, rivaroxban, apixaban or edoxaban) within 48 hrs (or 72 hours if patient has renal impairment – i.e. estimated glomerular filtration rate of <30ml/min), inherited bleeding disorder (i.e. any inherited clotting factor deficiencies, or platelet disorders), Pregnancy, Known or suspected allergy to FFP, or LG-octaplas, or PCC, Known or suspected allergy to heparin, Sodium citrate dihydrate, sodium dihydrogenphosphate dihydrate and Glycine, History of Heparin-induced thrombocytopenia, Individuals who have IgA deficiency with known antibodies against IgA, Documented venous thromboembolism in the last three months, Documented antiphospholipid syndrome, Severe protein S deficiency, Patients who are likely to go on Extracorporeal Membrane Oxygenation (ECMO) after cardiac surgery, Any other reasons the investigator may consider the patient to be ineligible for the study, and Participation in another clinical trial, where the patient has received IMP in the last 3 months

- 1. Data Collection:

***Screening****:* Baseline data (age, gender, ethnicity), type of admission (i.e. emergency or elective) and type of cardiac surgery, previous medical history, drug history, particularly antiplatelets or anticoagulants, QOL questionnaire (EQ5D), urine pregnancy test will be offered to women of child-bearing age (<50 years old)

***Randomisation****:* Standard laboratory tests as part of routine care, amount of blood component given pre-randomisation, amount of intravenous fluid administered pre-randomisation, date and time when study drugs started and completed, and research blood samples (15mL) pre-administration of intervention

***1-hour post intervention****:* Standard laboratory tests as part of routine care, and research blood samples (15mL) one hour after administration of intervention

***24-hours after surgery****: a*mount of blood lost through the chest drains, all blood components received (RBC, RBC, FFP, Platelets and cryoprecipitate) after randomisation excluding intervention, any other haemostatic agents (such as recombinant Factor VIIa, fibrinogen concentrate, cell salvage), standard laboratory tests as part of routine care and research blood samples (15mL)

**The following clinical data/complications were collected at 24hrs, *7, 14, 21 and 30 days, or discharge, or death (whichever was first)*:**

- Admission to ITU (level 3); High Dependency units (Level 2) and number of days in ITU/HDU
- Organ failure - i.e. acute lung injury, acute respiratory distress syndrome, cardiovascular failure; cardiac ischaemia or infarction; cardiac arrest, renal failure, liver failure
- Thrombosis (arterial and venous thrombosis) as confirmed by radiological testing (ultrasound, CT scans etc) as part of routine care.
- Acute transfusion reaction
- Infections such as pneumonia; surgical site infection; sepsis or any other infections
- Duration of organ support (i.e. ventilatory support, cardiovascular support, and renal replacement therapy)
- Duration of hospital stay
- Mortality

**The following clinical data/complications were collected a*t 90 days, or death – whichever is first***

- Mortality
- Re-hospitalisation
- Thromboembolic event (arterial and venous)
- Number of days alive and out of hospital since operation
- QOL questionnaire
  1. General Definitions for Adverse Events and Serious Adverse Events -

| **Term** | **Definition** |
| --- | --- |
| Adverse Event (AE) | Any untoward medical occurrence in a participant to whom a medicinal product has been administered, including occurrences which are not necessarily caused by or related to that product. |
| Adverse Reaction (AR) | An untoward and unintended response in a participant to an investigational medicinal product which is related to any dose administered to that participant.  The phrase "response to an investigational medicinal product" means that a causal relationship between a trial medication and an AE is at least a reasonable possibility, i.e. the relationship cannot be ruled out.  All cases judged by either the reporting medically qualified professional or the Sponsor as having a reasonable suspected causal relationship to the trial medication qualify as adverse reactions. |
| Serious Adverse Event (SAE) | A serious adverse event is any untoward medical occurrence that:   - Results in death. - Is life-threatening. - Requires inpatient hospitalisation or prolongation of existing hospitalisation - Results in persistent or significant disability/incapacity. - Consists of a congenital anomaly or birth defect.   Other ‘important medical events’ may also be considered serious if they jeopardise the participant or require an intervention to prevent one of the above consequences.  NOTE: The term "life-threatening" in the definition of "serious" refers to an event in which the participant was at risk of death at the time of the event; it does not refer to an event which hypothetically might have caused death if it were more severe. |
| Serious Adverse Reaction (SAR) | An adverse event that is both serious and, in the opinion of the reporting Investigator, believed with reasonable probability to be due to one of the trial treatments, based on the information provided. |
| Suspected Unexpected Serious Adverse Reaction (SUSAR) | A serious adverse reaction, the nature and severity of which is not consistent with the information about the medicinal product in question set out in the Reference Safety Information (RSI):   - In the case of a product with a marketing authorisation, in the summary of product characteristics (SmPC) for that product. - In the case of any other investigational medicinal product, in the investigator’s brochure (IB) relating to the trial in question. |

- 1. Laboratory methods

To assess haemostatic capacity, 15 mL whole blood samples (drawn into five 3mL BD Vacutainers,^TM^ [BD Diagnostics, Oxford, UK] each containing 0.27ml of 0.109 M buffered tri-sodium citrate at a ratio of 1 part anticoagulant to 9 parts blood) were taken on randomised subjects at three-time points: 1) prior to intervention; 2) within one hour of intervention being completed; and 3) 24 hours after intervention administration. Within 4 hours of sample collection platelet poor plasma was prepared by double centrifugation at ambient temperature (2000g for 12 minutes), using laboratory standard operating procedure. Aliquots of platelet poor plasma were stored at -70°C+/-10°C and on the day of assay aliquoted samples were thawed at 37°C.

The following assays were performed on a Sysmex CS-5100 analyser (Sysmex UK, Milton Keynes, UK) using standard protocols. All reagents, calibrators and controls were supplied by Sysmex UK (Milton Keynes, UK) except where indicated:

1. Calibrated against Siemens Standard Plasma
2. Calibrated against Technoclone Coagulation Reference Plasma (Pathway Diagnostics, Dorking, UK)
3. Calibrated against Hyphen Biomed Biophen UFH Calibrator Plasmas

*Screening assays:* Prothrombin Time (Siemens Dade Innovin), Activated Partial Thromboplastin Time: (Siemens Dade Actin FS) and Clauss fibrinogen (Siemens Dade Thrombin)

One-stage factor assays:

- Factor II, V, VII and X: Siemens Dade Innovin and Siemens factor deficient plasmas^1^
- Factor VIII, IX, XI and XII: Siemens Dade Actin FS and Siemens factor deficient plasmas^1^
- High molecular weight kininogen activity: Siemens Pathromtin SL and Technoclone Fitzgerald Trait Plasma (Pathway Diagnostics, Dorking, UK)^2^
- Prekallikrein activity: Siemens Pathromtin SL and Hyphen Biomed Prekallikrein deficient plasma^2^

*Chromogenic assays:*

- Heparin anti-Xa activity: Hyphen Biomed Biophen Heparin LRT^3^
- Heparin anti-IIa activity: Hyphen Biomed Biophen Anti-IIa (two stage heparin assay)^3^
- C1-inhibitor activity: Siemens Berichrom C1-inhibitor^1^
- Antithrombin activity: Hyphen Biomed Biophen Antithrombin (Xa) LRT^1^
- Protein C activity: Hyphen Biomed Biophen Protein C LRT^1^
- Alpha-2-antiplasmin activity: Hyphen Biomed Biophen α2-antiplasmin LRT^1^
- Plasminogen activity: Hyphen Biomed Biophen Plasminogen LRT^1^
- Factor XIII activity: Hyphen Biomed Biophen Factor XIII^1^

*Latex immunoassays:*

- D-Dimer: Siemens INNOVANCE D-Dimer
- Von Willebrand factor antigen: Hyphen Biomed Biophen VWF LRT^1^
- Von Willebrand factor activity: Siemens INNOVANCE vWF Ac^1^
- Free protein S antigen: Hyphen Biomed Biophen Free PS^1^

*Enzyme linked immunosorbent assays [ELISA]*

All ELISA assays were performed manually and results obtained by reading on a BioTek ExL500 plate reader attached to Gen5 software:

- Tissue plasminogen activator antigen: Hyphen Biomed ZYMUTEST tPA antigen (Quadratech, Lewes, UK)
- Tissue factor activatable fibrinolysis inhibitor: Hyphen Biomed ZYMUTEST (activatable) TAFI (Quadratech, Lewes, UK)
- Prothrombin factor 1+2: Siemens ENZYGNOST F 1+2 (monoclonal)
- Thrombin-antithrombin (TAT) complex: Siemens ENZYGNOST TAT micro
- Plasmin-antiplasmin (PAP) complex: Fine Test Human PAP ELISA kit (Quadratech, Lewes, UK)
- Soluble endothelial protein C Receptor (sEPCR): Fine Test Human SEPCR/PROCR ELISA kit (Quadratech, Lewes, UK)
- Thrombomodulin: Fine Test Human TM ELISA kit (Quadratech, Lewes, UK)
- Tissue factor: Hyphen Biomed ZYMUTEST Tissue Factor (full length) (Quadratech, Lewes, UK)

*Thrombin generation*: Thrombin generation was performed using the Calibrated Automated Thrombogram Fluoroskan system (Thrombinoscope BV, Maastricht, The Netherlands) in conjunction with the manufacturer’s PPP reagents (Diagnostica Stago UK, Theale, UK), which gave reaction concentrations of 5pM tissue factor and 4µM phospholipid. The following parameters of the thrombin generation curve were measured: lag time; time to peak; peak thrombin; and area under the curve, known also as the endogenous thrombin potential.

| **Table S1.** | Clotting factor results: Change from baseline at 1 hour and 24 hours after intervention | | | | | | | | |  | |  |
| --- | --- | --- | --- | --- | --- | --- | --- | --- | --- | --- | --- | --- |
|  |  | **FFP** | | **PCC** | | | |  | | | |  |
| **Clotting assays** | **time** | **MEDIAN [IQR]** | **n** | **MEDIAN [IQR]** | | | **n** | **Effect size** | | | | **(Confidence Interval)** |
| **PT** | 1 hour | -4 [-6.1 to -2.1] | 19 | -2.6 [-4.85 to -1.3] | | | 16 | 1 | | | | ( -1.94 to 3.94 ) |
|  | 24 hours | -4.45 [-6.6 to -2.75] | 16 | -3.9 [-6.1 to -1.9] | | | 14 | 1 | | | | ( -2.50 to 4.50 ) |
| **APTT** | 1 hour | -12.9 [-40.7 to -6.4] | 19 | -4.2 [-16.8 to .1] | | | 15 | 8.7 | | | | ( -13.21 to 30.61 ) |
|  | 24 hours | -30.55 [-49.4 to -7.1] | 16 | -7.9 [-23.9 to -6.1] | | | 13 | 28.1 | | | | ( -1.19 to 57.39 ) |
| **Fibrinogen** | 1 hour | .34 [.2 to .6] | 19 | .01 [-.1 to .26] | | | 17 | -0.33 | | | | ( -0.59 to -0.07 ) |
|  | 24 hours | 1.85 [1.19 to 2.57] | 16 | 1.7 [1.06 to 2.08] | | | 14 | -0.12 | | | | ( -1.04 to 0.80 ) |
| **D-dimer** | 1 hour | -.13 [-.93 to .05] | 19 | -.04 [-.18 to .27] | | | 17 | 0.09 | | | | ( -0.31 to 0.49 ) |
|  | 24 hours | -.08 [-.68 to -.01] | 16 | -.04 [-.36 to .05] | | | 14 | 0.05 | | | | ( -0.50 to 0.60 ) |
| **Factor II** | 1 hour | 11.4 [4.5 to 16.3] | 19 | 22.4 [20.3 to 27.3] | | | 17 | 11 | | | | ( 2.86 to 19.14 ) |
|  | 24 hours | 21.1 [3.55 to 28.8] | 16 | 21.45 [11.2 to 31.9] | | | 14 | -4.5 | | | | ( -22.82 to 13.82 ) |
| **Factor V** | 1 hour | 26.2 [12.8 to 40.3] | 19 | .3 [-2 to 6.6] | | | 17 | -25.9 | | | | ( -38.40 to -13.40 ) |
|  | 24 hours | 59.3 [28.2 to 82.25] | 16 | 33.45 [15.3 to 55.9] | | | 14 | -19 | | | | ( -51.60 to 13.60 ) |
| **Factor VII** | 1 hour | 16.7 [8 to 28.2] | 19 | 16.4 [12.5 to 23.8] | | | 17 | -0.3 | | | | ( -10.49 to 9.89 ) |
|  | 24 hours | -4.55 [-10.7 to 17.5] | 16 | 8.2 [-14.1 to 13.5] | | | 14 | 11.1 | | | | ( -9.65 to 31.85 ) |
| **Factor VIII** | 1 hour | 8.4 [-14.3 to 40.8] | 19 | 1 [-7.3 to 31.7] | | | 17 | -7.4 | | | | ( -37.09 to 22.29 ) |
|  | 24 hours | 106.1 [79.65 to 121.05] | 16 | 87.45 [57.1 to 127.4] | | | 14 | -10.7 | | | | ( -60.80 to 39.40 ) |
| **Factor IX** | 1 hour | 7.2 [-8.6 to 24.9] | 19 | 14.9 [3.4 to 21] | | | 17 | 7.7 | | | | ( -10.73 to 26.13 ) |
|  | 24 hours | 28.35 [6.5 to 40.2] | 16 | 27.1 [4 to 40.4] | | | 14 | -0.3 | | | | ( -24.02 to 23.42 ) |
| **Factor X** | 1 hour | 11.6 [4.8 to 18.1] | 19 | 20.9 [17.2 to 26.5] | | | 17 | 9.3 | | | | ( 0.71 to 17.89 ) |
|  | 24 hours | 22.25 [6.3 to 27] | 16 | 22.45 [9.3 to 29.8] | | | 14 | -3.4 | | | | ( -20.50 to 13.70 ) |
| **Factor XI** | 1 hour | 8.4 [3.1 to 27.1] | 19 | | 0 [-3.9 to 6.8] | | 17 | -8.4 | | | | ( -20.90 to 4.10 ) |
|  | 24 hours | 20.75 [9.4 to 38.3] | 16 | | 5.65 [0 to 21.5] | | 14 | -16.4 | | | | ( -35.84 to 3.04 ) |
| **Factor XII** | 1 hour | 10.5 [1.5 to 17.4] | 19 | | -2.2 [-7.2 to 3.5] | | 17 | -12.7 | | | | ( -24.46 to -0.94 ) |
|  | 24 hours | 21.5 [-.5 to 32.7] | 16 | | 10.75 [.3 to 24.2] | | 14 | -13.1 | | | | ( -34.34 to 8.14 ) |
| **Factor XIII** | 1 hour | 10.5 [2.7 to 19.1] | 19 | | -1.8 [-5.5 to 3.2] | | 17 | -12.3 | | | | ( -22.26 to -2.34 ) |
|  | 24 hours | 7.05 [4.1 to 14.3] | 16 | | .9 [-16.3 to 12.3] | | 14 | -9.1 | | | | ( -24.02 to 5.82 ) |
| **VWF antigen** | 1 hour | 1.7 [-14.9 to 28.6] | 19 | | 20.35 [-5.75 to 39] | | 16 | 18.9 | | | | ( -11.96 to 49.76 ) |
|  | 24 hours | 69.65 [5.45 to 119.25] | 16 | | 83.95 [53.9 to 124.4] | | 14 | 16.6 | | | | ( -41.51 to 74.71 ) |
| **VWF activity** | 1 hour | 20.1 [-.9 to 42.7] | 19 | | 17.35 [-.5 to 88.6] | | 16 | 0 | | | | ( -52.59 to 52.59 ) |
|  | 24 hours | 87.65 [29.55 to 129.7] | 16 | | 101.6 [82.2 to 124.2] | | 14 | 9.7 | | | ( -50.45 to 69.85 ) | |
| **AT activity** | 1 hour | 9.9 [4.5 to 12.9] | 18 | | .5 [-2 to 3.7] | | 16 | -9 | | | ( -13.96 to -4.04 ) | |
|  | 24 hours | 22.25 [8.1 to 29.4] | 16 | | 14.75 [4.7 to 18.1] | | 14 | -7.5 | | | ( -18.34 to 3.34 ) | |
| **PC activity** | 1 hour | 11.2 [5.8 to 21] | 19 | | 19.9 [9.1 to 29] | | 17 | 8.7 | | | ( -0.72 to 18.12 ) | |
|  | 24 hours | 21.25 [14.05 to 36.65] | 16 | | 18.85 [-.2 to 33.8] | | 14 | -5.1 | | | ( -24.55 to 14.35 ) | |
| **FPS antigen** | 1 hour | 8.35 [4.4 to 11.7] | 18 | | 13 [6.55 to 16.05] | | 16 | 5.1 | | | ( -0.03 to 10.23 ) | |
|  | 24 hours | 15.6 [-2.1 to 24.55] | 16 | | 12.2 [4.6 to 19.3] | | 13 | -3.1 | | | ( -16.88 to 10.68 ) | |
| **TAT** | 1 hour | 0 [0 to .3] | 16 | | 0 [0 to .2] | | 16 | 0 | | | ( -3.79 to 3.79 ) | |
|  | 24 hours | -5 [-12.4 to 0] | 14 | | -2.4 [-12.1 to 0] | | 13 | 1.6 | | | ( -8.85 to 12.05 ) | |
| **HMWK** | 1 hour | 3.15 [-4.1 to 20] | 16 | | 10.9 [-2.6 to 23.3] | | 15 | 7 | | | ( -10.29 to 24.29 ) | |
|  | 24 hours | 6.3 [-7.9 to 21.4] | 14 | | 14.6 [-9.75 to 24.45] | | 12 | 8.4 | | | ( -17.30 to 34.10 ) | |
| **PRK** | 1 hour | 10.3 [-2.85 to 18.1] | 16 | | -4 [-12 to 14.5] | | 15 | -17.4 | | | ( -35.70 to 0.90 ) | |
|  | 24 hours | 5.55 [-6.4 to 25.7] | 14 | | -2.8 [-34.75 to 13.3] | | 12 | -12.3 | | | ( -44.12 to 19.52 ) | |
| **C1-inhibitor** | 1 hour | 7.2 [-6.4 to 13.1] | 19 | | 2.3 [-7.55 to 7.1] | | 16 | -7.4 | | | ( -19.62 to 4.82 ) | |
|  | 24 hours | 34.15 [8.1 to 45.95] | 16 | | 24.2 [10.9 to 40.2] | | 14 | -4.6 | | | ( -29.15 to 19.95 ) | |
| **α_2_-antiplasmin** | 1 hour | 8.4 [4.2 to 16.7] | 19 | | -1 [-2.9 to 7.2] | | 17 | -9.4 | | | ( -17.15 to -1.65 ) | |
|  | 24 hours | 34.85 [16.5 to 43.25] | 16 | | 23.95 [6.3 to 36] | | 14 | -11.2 | | | ( -35.82 to 13.42 ) | |
| **Plasminogen** | 1 hour | 6.7 [1.3 to 11.7] | 19 | | -1 [-4.2 to 1] | | 17 | -7.7 | | | ( -13.31 to -2.09 ) | |
|  | 24 hours | 16.35 [8.25 to 22.85] | 16 | | 2.6 [-3.8 to 10.5] | | 14 | -12.1 | | | ( -22.05 to -2.15 ) | |
| **tPA:Ag** | 1 hour | -.3 [-1.7 to 1.1] | 19 | | 0 [-1.35 to 1.1] | | 16 | 0.3 | | | ( -1.10 to 1.70 ) | |
|  | 24 hours | .2 [-1.4 to 3.95] | 16 | | -1.5 [-3.4 to 0] | | 13 | -1.8 | | | ( -5.36 to 1.76 ) | |
| **Prothrombin F.1+2** | 1 hour | 13 [0 to 219.5] | 16 | | 0 [-233.5 to 137] | | 16 | -26 | | | ( -244.38 to 192.38 ) | |
|  | 24 hours | -524.5 [-920 to -317] | 14 | | -651 [-934 to -398] | | 13 | -153 | | | ( -542.95 to 236.95 ) | |
| **PAP** | 1 hour | 104 [-34 to 336] | 18 | | -54 [-139 to 38.5] | | 16 | -157 | | | ( -386.64 to 72.64 ) | |
|  | 24 hours | 179 [-73 to 391] | 15 | | -49 [-220 to 320] | | 13 | -228 | | | ( -593.96 to 137.96 ) | |
| **TAFI** | 1 hour | 9 [3 to 16] | 19 | | 4 [-1 to 7] | | 16 | -5 | | | ( -11.00 to 1.00 ) | |
|  | 24 hours | 13 [7.5 to 21.5] | 16 | | 8 [-2 to 11] | | 13 | -5 | | | ( -15.96 to 5.96 ) | |
| **Thrombomodulin** | 1 hour | 100 [30 to 524] | 18 | | 183.5 [67 to 406.5] | | 16 | 146 | | | ( -131.72 to 423.72 ) | |
|  | 24 hours | 38 [-67 to 251] | 15 | | 70 [3 to 169] | | 13 | 32 | | | ( -162.33 to 226.33 ) | |
| **Tissue Factor** | 1 hour | .8 [-.7 to 8.4] | 17 | | | .05 [-2.05 to 1.35] | | | 16 | -0.8 | | ( -5.79 to 4.19 ) |
|  | 24 hours | 1 [-.5 to 2.2] | 15 | | | 0 [-2.1 to .4] | | | 13 | -1 | | ( -3.21 to 1.21 ) |
| **sEPCR** | 1 hour | 0 [0 to 749] | 18 | | | 0 [-1319 to 713.5] | | | 16 | 0 | | ( -1129.98 to 1129.98 ) |
|  | 24 hours | 0 [-1167 to 0] | 15 | | | -362 [-2189 to 167] | | | 13 | -362 | | ( -2202.82 to 1478.82 ) |
| **TG- ETP** | 1 hour | 350 [-237 to 667] | 17 | | | 159 [-352 to 714] | | | 17 | -191 | | ( -765.84 to 383.84 ) |
|  | 24 hours | 581.5 [112 to 961] | 14 | | | 686 [116 to 1407] | | | 14 | 27 | | ( -763.25 to 817.25 ) |
| **TG- Peak thrombin** | 1 hour | 88 [-48 to 118] | 17 | | | 6 [-57 to 64] | | | 17 | -82 | | ( -194.68 to 30.68 ) |
|  | 24 hours | 82.5 [-29 to 166] | 14 | | | 53 [-7 to 223] | | | 14 | -14 | | ( -141.40 to 113.40 ) |
| **TG- lag time** | 1 hour | -1.33 [-5.61 to .44] | 17 | | | -.45 [-.88 to .33] | | | 17 | 0.88 | | ( -2.51 to 4.27 ) |
|  | 24 hours | -1.69 [-5.61 to 1.11] | 14 | | | .34 [-.22 to 2] | | | 14 | 2.78 | | ( -0.75 to 6.31 ) |
| **TG- time to peak** | 1 hour | -2.83 [-9.56 to .61] | 17 | | | .67 [-.89 to 2] | | | 17 | 3.5 | | ( -0.88 to 7.88 ) |
|  | 24 hours | -3.44 [-9.33 to 1.89] | 14 | | | 1.11 [-.67 to 3.22] | | | 14 | 4.71 | | ( -0.97 to 10.39 ) |

Abbreviations: PT: prothrombin time; APTT: activated partial thromboplastin time; AT: antithrombin; PC: protein C; FPS: free protein S; TAT: thrombin-antithrombin complex; HMWK: high molecular weight kininogen activity; PRK: prekallikrein activity; tPA:Ag: tissue plasminogen activator antigen; PAP: plasmin-antiplasmin complex; TAFI: tissue activatable fibrinolysis inhibitor; sEPCR: soluble endothelial protein C receptor; TG: thrombin generation; ETP: endogenous thrombin potential

**Figure S1: Pooled Safety Outcomes updated from the systematic review^5^**

**Mortality: in hospital or at 30 days**


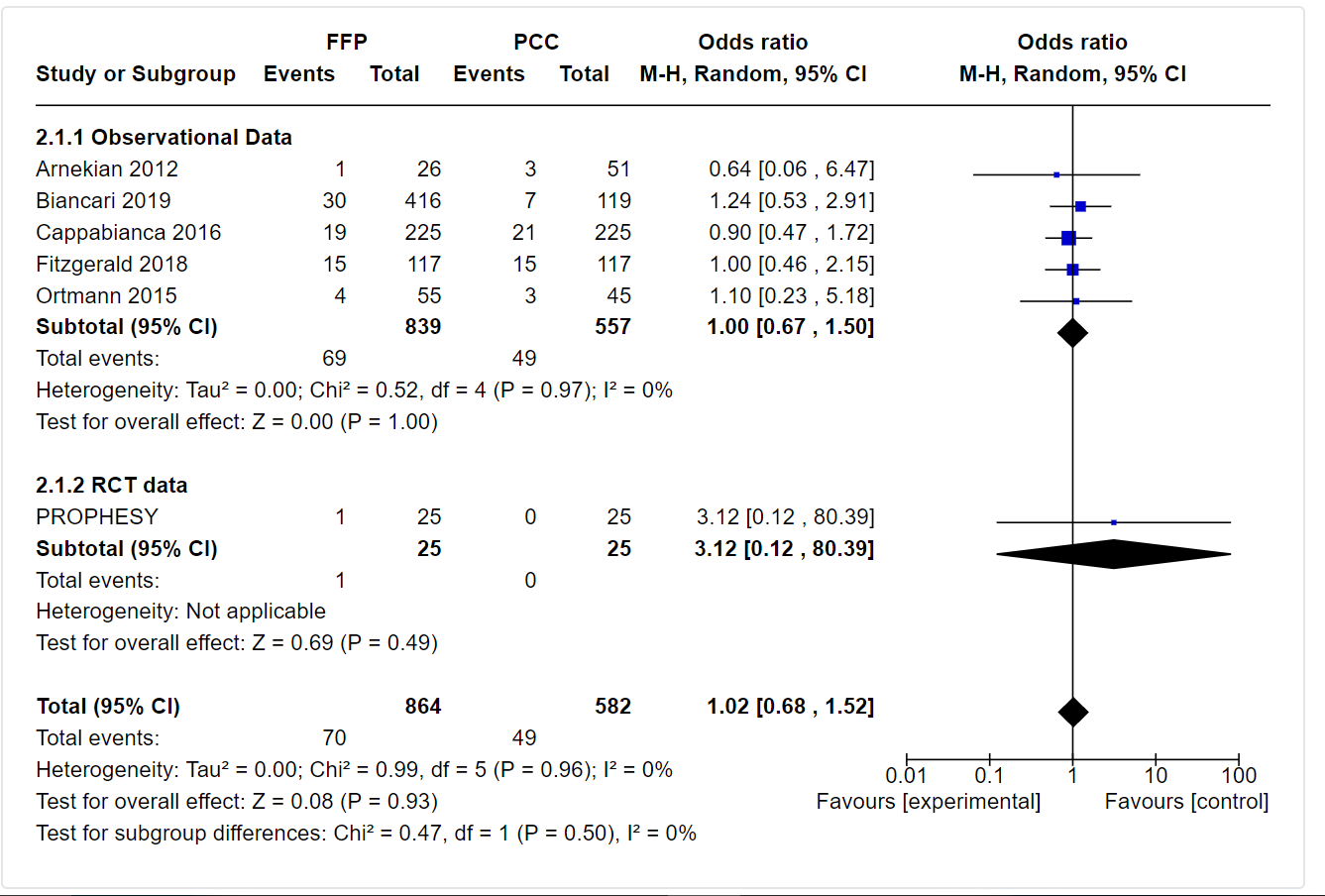


**Stroke**


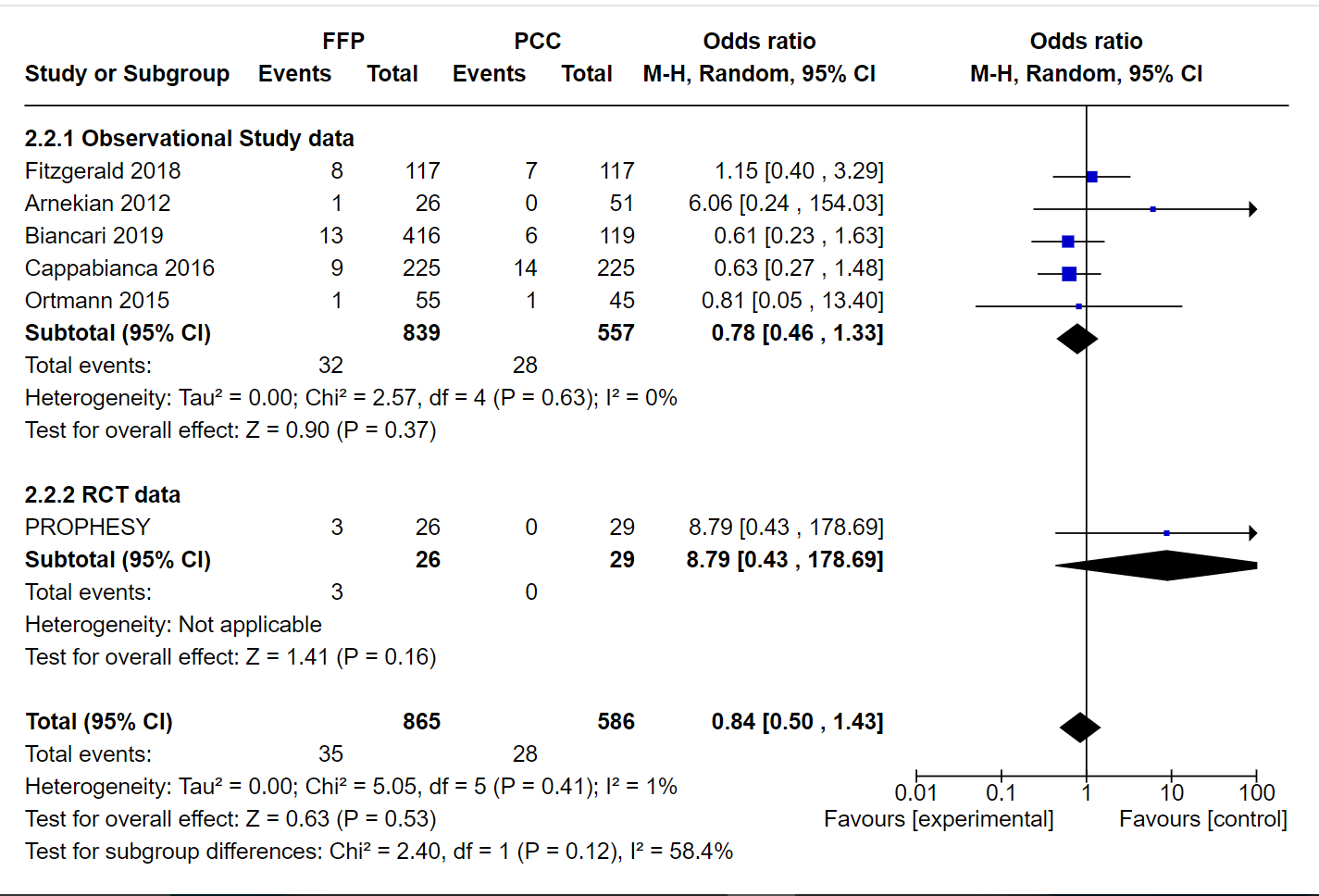


**FigureS 2:** Thromboelastography-based protocol
